# Olmesartan medoxomil reverses glomerulosclerosis in renal tissue induced by myocardial infarction without changes in renal function

**DOI:** 10.3892/etm.2014.1695

**Published:** 2014-04-25

**Authors:** XIAO-MEI LU, YU-NAN JIN, LING MA

**Affiliations:** Department of Pathophysiology, College of Basic Medical Sciences, China Medical University, Shenyang, Liaoning 110001, P.R. China

**Keywords:** mouse, myocardial infarction, olmesartan medoxomil

## Abstract

The aim of the present study was to investigate the effect of olmesartan medoxomil (OLM) on renal injury in mice with myocardial infarction (MI). A total of 33 male C57/BL/6 mice were divided into a sham surgery group (SHAM group), MI group (MI group) and OLM treatment group (OLM group). Experimental MI models were established in the mice of the MI and OLM groups by coronary artery ligation, and the mice in the OLM group were fed a daily dose of 10 mg/kg OLM for eight weeks. The results showed that MI induced a reduction in cardiac function and an increase in systolic blood pressure. In addition, increased periodic acid-Schiff (PAS) positive staining, combined with increased levels of angiotensin II (Ang II) in the plasma and kidneys, and increased expression levels of renin, angiotensin II type 1 receptor (AT1R) and angiotensinogen (AGT) in the kidney tissues was observed compared with those in the SHAM group. OLM treatment attenuated the injury by reducing the systolic blood pressure and PAS positive staining, and decreasing the expression levels of Ang II, renin, AT1R and AGT in the kidney compared with those in the MI group. It may be concluded that MI activates the intrarenal renin-angiotensin system and leads to glomerulosclerosis, and that OLM protects the kidney by inhibiting the effects of Ang II.

## Introduction

Heart failure (HF) is a complex syndrome, characterized by symptoms associated with inadequate perfusion of organs and tissues during exercise, and represents the common pathway for the majority of primary cardiovascular diseases (1). Progressive aging of the population, together with the significant reduction in the rate of mortalities due to cardiovascular diseases, have been accompanied by a marked increase in the prevalence of HF, and HF is now considered a major public health problem (2).

Patients with myocardial infarction (MI) that has progressed to a late stage develop chronic HF. Chronic HF with renal insufficiency, which is known as cardiorenal syndrome (CRS), has gradually become a focus of drug discovery efforts (3). Congestive HF is a progressive disorder that leads to intense intrarenal vasoconstriction, elevated renal vascular resistance, reduced renal blood flow (4), progressive kidney damage and renal insufficiency in the later stages (5). The incidence of chronic cardiovascular disease is increasing and, due to the surgical treatment of numerous patients in the acute phase of the disease, the number of patients with progression of the disease to end-stage has also increased markedly; such patients often present with renal insufficiency and CRS manifestations (6). The pathogenesis of CRS is complex and studies have identified that excessive activation of the renin-angiotensin system (RAS) is an important risk factor (7). The RAS produces angiotensin II (Ang II) and participates in damaging renal hemodynamic and non-hemodynamic effects. The non-hemodynamic effects of the RAS, including the promotion of oxidative stress, are mainly generated by Ang II local to an organ (8). Ang II initiates intracellular oxidative stress through the protein kinase C pathway, stimulates the NADH/NADPH oxidase system, and produces large amounts of reactive oxygen species resulting in kidney tissue damage (9).

The mouse model of MI is useful model for studying the intrarenal abnormalities in congestive HF (10). An *in vitro* and *in vivo* study has shown that olmesartan medoxomil (OLM) blocks Ang II-induced physiological activity (11). Thus, the present study used a left anterior descending artery (LAD) ligation MI model and olmesartan intervention to observe activation of the RAS of the kidney and the protective effect of olmesartan against renal injury.

## Materials and methods

### Animal preparation

Ten-week-old male C57/BL/6 mice were used in the experiments. The mice were housed under climate-controlled conditions with a 12-h light/dark cycle and were provided with standard food and water *ad libitum*. All experimental procedures were performed under the guidelines for the care and use of animals as established by the China Medical University (Shenyang, China). The mice were divided into three groups: The sham surgery (SHAM) group, MI group and OLM treatment group. The surgical protocol was performed similar to methods described previously (12), Briefly, the mice were anesthetized by 50 mg/kg phenobarbital sodium, then the mice were orally intubated with polyethylene tubing and connected to a rodent ventilator (type 845; Harvard Apparatus Ltd., Kent, UK). Positive-pressure artificial respiration was started immediately with room air, using a volume of 1.5 ml/100 g bodyweight at a rate of 90 strokes/min to maintain the normal PCO_2_ (40 mmHg), PO_2_ (100 mmHg) and pH (7.20) parameters. The chest was opened by a left thoracotomy, followed by sectioning of the fourth and fifth ribs. The LAD was visualized and ligated with 7-0 silk suture. The chest wall was closed and the mice were allowed to recover in a temperature-controlled area. The mice were included in the MI group if there was a transmural left ventricular (LV) scar at autopsy obliterating ≥25% LV muscle. The mice in the OLM treatment group were fed a daily dose of 10 mg/kg OLM for eight weeks. Sham surgery group mice underwent the same surgery minus the coronary artery ligation.

### Determination of heart rate (HR), systolic blood pressure (SBP) and cardiac function

One day prior to sacrifice of the animals, an intelligent non-invasive blood pressure monitor (Kent Scientific Corporation, Torrington, CT, USA) was used to measure the SBP and HR in the awake state. Transthoracic echocardiography was performed prior to and eight weeks after the surgery using a HP 5500 imaging system (Hewlett-Packard Co., Palo Alto, CA, USA) equipped with a 15 MHz probe. The M-mode cursor was positioned perpendicular to the anterior wall in order to measure the interventricular septum, and the LV end-diastolic and end-systolic diameters (LVDd and LVDs, respectively) at the level of the papillary muscles below the mitral valve tip, and the LV ejection fraction (EF) and fractional shortening (FS) were calculated.

### Determination of blood urea nitrogen (BUN) levels, serum creatinine (SCr) and urinary protein and albumin concentrations

Following anesthesia, decapitation of the animals was used, trunk blood was collected in chilled tubes and processed for measurements of the plasma BUN levels and SCr. The urinary protein and albumin concentrations were measured using an ELISA kit (Exocell Inc., Philadelphia, PA, USA) from a one-day timed urine collection at eight weeks after the surgery.

### Determination of plasma and kidney Ang II levels

Following decapitation, trunk blood was collected from the mice in chilled tubes and the plasma was separated and stored. Simultaneously, 50 mg kidney specimens were homogenized in an ice bath and centrifuged to obtain supernatants. The Ang II levels in the plasma and renal tissue homogenate were detected with an radioimmunoassay kit according to the manufacturer’s instructions (Nanjing Jiancheng Bioengineering Institute, Nanjing, China).

### Determination of the renal pathological changes by periodic acid-Schiff (PAS) staining

The animals were sacrificed by decapitation, followed by immediate organ collection for histological analysis. Fresh kidney sections were fixed in 10% buffered formalin and embedded in paraffin, and 4-μm sections were stained using the PAS method. A pathologist blind to the group assignments analyzed the samples and determined the levels of injury.

### Determination of renin, angiotensin II type 1 receptor (AT1R) and angiotensinogen (AGT) expression levels in the kidney tissues by quantitative reverse transcription-polymerase chain reaction (PCR)

The mRNA expression levels of glyceralde-hyde-3-phosphate dehydrogenase (GAPDH), renin, AT1R and AGT were analyzed by quantitative PCR using a Light Cycler Fast Start DNA Master SYBR Green I kit (Applied Biosystems, Foster City, CA, USA) in ABI PRISM 7900T real-time PCR system (Applied Biosystems). The primer sequences are shown in Table I. For the PCR, the conditions were as follows: 30 sec at 94°C; 40 cycles at 94°C for 5 sec, 55°C for 5 sec, and 72°C for 15 sec. All data are expressed as the relative differences following normalization to the GAPDH expression levels.

### Statistical analysis

Data are expressed as mean ± standard error of the mean of the number of animals. P<0.05 was considered to indicate a statistically significant difference. Multiple comparisons between the experimental groups were performed by one-way analysis of variance with Tukey’s post hoc test.

## Results

### General state and cardiac function changes in each group

Eight weeks after the surgery, all animals in the SHAM group were alive and generally in good condition; in MI group, three mice had died due to severe HF and the remaining mice exhibited varying degrees of shortness of breath, wheezing, and eating and activity reduction; and the mice in the OLM group also showed slightly reduced eating and activity. The mice in the MI and OLM groups exhibited anatomical visible LV chamber and liver enlargement, lung congestion, pleural effusion and ascites. The transthoracic echocardiography results showed that the cardiac infarction lead to LV enlargement and systolic dysfunction when compared with the results of the sham surgery mice (P<0.05). When compared with those of the MI mice, the mice in the OLM group had less LV enlargement (measured as the LVDd and LVDs) and less LV systolic dysfunction (evaluated using the EF and FS). The MI group also exhibited increased SBP compared with that of the SHAM group, while in the OLM group the SBP decreased compared with that in the MI group. These results are shown in Table II.

### Renal injury and renal insufficiency in each group

In this study, the serum BUN levels, SCr, and urinary protein and albumin concentrations were examined and the results are summarized in Table III. Cardiac infarction lead to slightly increased serum BUN levels, SCr, and urinary protein and albumin concentrations compared with those of the SHAM group (P>0.05), and these increases were slightly attenuated in the OLM group (P>0.05), but no statistical significance was identified.

### Plasma and kidney Ang II levels in each group

MI induced increased Ang II levels in the plasma and kidney tissues compared with those in the SHAM group (P<0.05). However, compared with those of the MI group, the renal Ang II levels were significantly reduced, while the plasma levels were significantly increased in the OLM group (P<0.05). The results are shown in Table III.

### Renal pathological changes shown by PAS staining

**Figure 1:**
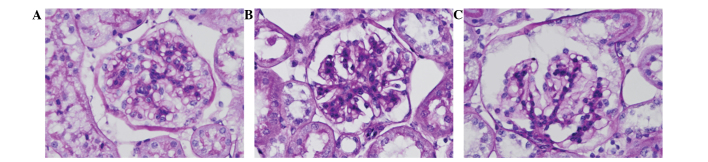
Representative PAS staining results of each group (magnification, ×400). (A) SHAM; (B) MI and (C) OLM group. PAS, periodic acid-Schiff; SHAM, sham surgery; MI, myocardial infarction; OLM, olmesartan medoxomil.

In the SHAM group, the PAS staining indicated normal glomerular and tubular structures, renal tubular epithelial cells arranged in neat rows and no inflammatory cell infiltration in the stroma. By contrast, in the MI group, glomerular mesangial and tubular lumen expansion and irregular thickening of the basement membrane were observed, and the renal interstitial PAS positive staining was markedly increased compared with that in the SHAM group. These changes were clearly reduced in the OLM group. The results are shown in Fig. 1.

### Intrarenal RAS activation during MI

The quantitative PCR results showed that the expression levels of renin, AT1R and AGT were increased in the MI group compared with those in the SHAM group (P<0.05). However, compared with those of the MI group, the levels were significantly reduced in the OLM group (P<0.05). The results are shown in Table IV.

## Discussion

Reduced cardiac mass following MI commonly leads to congestive HF in humans and experimental animal models (12). In patients with congestive HF, renal perfusion and function are often compromised, which is known as CRS (13,14). Cardiac and renal dysfunctions often occur simultaneously as they share causes and pathogenetic mechanisms. There is a close association between renal and cardiac function in acute and chronic diseases. Cardiovascular disease causes >50% of the mortalities of patients with renal failure, while poor renal function increases mortality in patients with HF (15). Traditionally, renal impairment has been attributed to renal hypoperfusion due to reduced cardiac output and systemic pressure. The hypovolemia leads to sympathetic activity, increased renin-angiotensin-aldosterone pathway activity and arginine-vasopressin release. In addition, as well as ischemia, inflammation and oxidative stress are considered the main determinants of cardiac and renal dysfunction (16).

During HF, renal perfusion reduction and RAS activation lead to progressive decline of renal function (17). An increasing number of studies have demonstrated that local RAS activation and the production of Ang II mediate numerous important roles, including cell growth, the long-term effects of cardiac and vascular hypertrophy, ventricular and vascular remodeling and even renal fibrosis (18,19). Existing studies have verified that the effects of Ang II associated with excessive activation of the kidney RAS include the generation of oxygen free radicals, the activation of NF-κB in fibroblast proliferation, inflammation and exaggerated extracellular matrix deposition (20,21,22). Excessive production of Ang II also affects glomerular mesangial cells and podocytes, leading to glomerulosclerosis and renal dysfunction (23).

The model of coronary ligation in mice was selected as a previous study has documented the typical hemodynamic changes of MI in this model (24). In the present study, the LAD of mice was occluded for eight weeks, and the mice exhibited significant LV enlargement and systolic dysfunction of the heart, accompanied by renal dysfunction which presented as slightly increased plasma BUN levels, SCr, and urinary protein and albumin concentrations with no statistical significance. The PAS staining results identified glomerulosclerosis accompanied by intrarenal RAS activation, which manifested as increased levels of Ang II in the plasma and kidney, and increased levels of renal renin, AT1R and AGT expression, which was consistent with the results of previous study (25).

There are four subtypes of Ang II receptor, but the physiological effect is usually mediated by the AT1R. Studies have shown that the AT1R is widely distributed in various cells of kidney tissue. The AT1R is usually combined with Ang II and results in renal vasoconstriction, reduced renal blood flow and increased glomerular pressure and mesangial extracellular matrix production, and simultaneously stimulates the production of certain growth factors (26). Angiotensin II type 2 receptors (AT2Rs) are mainly distributed in the glomerular afferent arterioles and mesangial cells, which usually dilate afferent arterioles, and inhibit the growth of mesangial cells and stimulate apoptosis. AT2Rs in the kidney stimulate the generation of NO, so these two types of receptor have opposite physiological roles (27). OLM is a prodrug, which is absorbed via the gastrointestinal tract and hydrolyzed to form olmesartan. Olmesartan is a selective AT1R antagonist, which antagonizes the effects of Ang II by selectively blocking the binding site at which Ang II and AT1R combine. The results of the present study showed that OLM combined with AT1R substantially suppressed excessive activation of intrarenal RAS, thereby blocking Ang II damage to the kidneys. The study also demonstrated that OLM significantly increased the plasma Ang II levels, which may be combined with AT2R in the circulation and play a protective role.

In conclusion, MI activates the intrarenal RAS and leads to glomerulosclerosis, and OLM protects the kidneys by inhibiting the effects of Ang II.

## Figures and Tables

**Table I:** Oligonucleotide primer sequences.

mRNA	5′ primer	3′ primer
Renin	GAGGCCTTCCTTGACCAATC	TGTGAATCCCACAAGCAAGG
AT1R	GGAAACAGCTTGGTGGTGATC	CTGAGACACGTGAGCAGGAAC
AGT	CACCCCTGCTACAGTCCATTG	GTCTGTACTGACCCCCTCCAG
GAPDH	ATCA TCA GCAA TGCCTCCTG	CCCTCCGACGCCTGCTT

AT1R, angiotensin II type 1 receptor; AGT, angiotensinogen; GAPDH, glyceraldehyde-3-phosphate dehydrogenase.

**Table II:** HR, SBP and echocardiography parameters of the mice (mean ± SEM; n=l0 per group).

Group	HR (beats/min)	SBP (mmHg)	IVS (mm)	LVDd (mm)	LVDs (mm)	FS (%)	EF (%)
SHAM	602.5±11.3	100.3±1.7	0.95±0.02	3.29±0.42	1.68±0.31	42.31±5.09	88.91±6.42
MI	598.7±12.7	119.1±2.3^a^	0.92±0.01	4.23±0.55^a^	3.59±0.52^a^	19.65±3.42^a^	59.16±4.73^a^
OLM	603.2±13.5	101.9±2.2^b^	0.91±0.01	4.01±0.48^a^	2.89±0.58^a^	29.69±3.41^a^	66.75±5.01^a^

a P<0.05 vs. SHAM group;

b P<0.05 vs. MI group.

HR, heart rate; SBP, systolic blood pressure; IVS, interventricular septum; LVDd, left ventricular end-diastolic diameter; LVDs, left ventricular end-systolic diameter; FS, fractional shortening; EF, ejection fraction; SHAM, sham surgery; MI, myocardial infarction; OLM, olmesartan medoxomil.

**Table III:** Serum BUN levels, SCr, urinary protein and albumin excretion concentration, and plasma and kidney Ang II levels (mean ± SEM; n=l0 per group).

Group	BUN (mmol/l)	SCr (μmol/l)	Urinary protein (mg/24 h)	Urinary albumin (μg/24 h)	Plasma Ang II (fmol/ml)	Kidney Ang II (fmol/g)
SHAM	18.4±4.9	49.6±6.8	25.5±0.7	17.7±0.9	64.3±20.1	27.4±11.9
MI	20.9±4.4	54.0±8.7	31.4±0.9	20.3±5.4	98.2±19.2^a^	171.3±19.1^a^
OLM	19.7±8.2	50.6±9.3	28.3±0.6	18.6±3.7	218.6±36.6^b^	98.7±11.5^b^

a P<0.05 vs. SHAM group;

b P<0.05 vs. MI group.

BUN, blood urea nitrogen; SCr, Serum creatinine; Ang II, angiotensin II; SHAM, sham surgery; MI, myocardial infarction; OLM, olmesartan medoxomil.

**Table IV:** Expression levels of renin, AT1R and AGT in the kidney tissue, detected by quantitative PCR (mean ± SEM; n=l0 per group).

Group	Renin	AT1R	AGT
SHAM	0.20±0.02	1.55±0.07	1.00±0.10
MI	1.31±0.07^a^	2.21±0.10^a^	1.74±0.07^a^
OLM	0.56±0.07^b^	1.84±0.07^b^	1.36±0.09^b^

a P<0.05 vs SHAM group;

b P<0.05 vs MI group.

AT1R, angiotensin II type 1 receptor; AGT, angiotensinogen; PCR, polymerase chain reaction; SHAM, sham surgery; MI, myocardial infarction; OLM, olmesartan medoxomil.

## References

[1] Wang L, Shi J, Zhang Y (2013). Influences of simvastatin on vascular endothelial function of patients with coronary heart disease complicated with congestive heart failure. Eur Rev Med Pharmacol Sci.

[2] Hayashi H, Fukuma N, Kato K, Kato Y, Takahashi H, Mizuno K (2013). Clinical backgrounds and the time course of sleep-disordered breathing in patients after myocardial infarction. J Nippon Med Sch.

[3] Longhini C, Molino C, Fabbian F (2010). Cardiorenal syndrome: still not a defined entity. Clin Exp Nephrol.

[4] Ronco C, Kaushik M, Valle R, Aspromonte N, Peacock WF (2012). Diagnosis and management of fluid overload in heart failure and cardio-renal syndrome: the ‘5B’ approach. Semin Nephrol.

[5] Ronco C (2010). Cardiorenal syndromes: definition and classification. Contrib Nephrol.

[6] Shchekochikhin D, Schrier RW, Lindenfeld J (2013). Cardiorenal syndrome: pathophysiology and treatment. Curr Cardiol Rep.

[7] Weir MR (2007). Effects of renin-angiotensin system inhibition on end-organ protection: can we do better?. Clin Ther.

[8] Bader M, Ganten D (2008). Update on tissue renin-angiotensin systems. J Mol Med (Berl).

[9] Plumb RD, El-Sherbeeny NA, Dixon LJ (2005). NAD(P)H-dependent superoxide production in platelets: the role of angiotensin II and protein kinase C. Clin Biochem.

[10] Parlakpinar H, Ozer MK, Cicek E, Cigremis Y, Vardi N, Acet A (2006). Renal damage in rats induced by myocardial ischemia/reperfusion: Role of nitric oxide. Int J Urol.

[11] Koike H, Sada T, Mizuno M (2001). In vitro and in vivo pharmacology of olmesartan medoxomil, an angiotensin II type AT1 receptor antagonist. J Hypertens Suppl.

[12] Venugopal J, Rajeswari R, Shayanti M, Sridhar R, Sundarrajan S, Balamurugan R, Ramakrishna S (2013). Xylan polysaccharides fabricated into nanofibrous substrate for myocardial infarction. Mater Sci Eng C Mater Biol Appl.

[13] Ronco C, McCullough P, Anker SD, Acute Dialysis Quality Initiative (ADQI) consensus group (2010). Cardio-renal syndromes: report from the consensus conference of the acute dialysis quality initiative. Eur Heart J.

[14] Shah BN, Greaves K (2010). The cardiorenal syndrome: a review. Int J Nephrol.

[15] Coresh J, Astor BC, Greene T, Eknoyan G, Levey AS (2003). Prevalence of chronic kidney disease and decreased kidney function in the adult US population: Third National Health and Nutrition Examination Survey. Am J Kidney Dis.

[16] Ritz E (2006). Heart and kidney: fatal twins?. Am J Med.

[17] Raizada V, Skipper B, Luo W, Griffith J (2007). Intracardiac and intrarenal renin-angiotensin systems: mechanisms of cardiovascular and renal effects. J Investig Med.

[18] Souza ÁP, Sobrinho DB, Almeida JF (2013). Angiotensin II type 1 receptor blockade restores angiotensin-(1–7)-induced coronary vasodilation in hypertrophic rat hearts. Clin Sci (Lond).

[19] Shao W, Seth DM, Prieto MC, Kobori H, Navar LG (2013). Activation of the renin-angiotensin system by a low-salt diet does not augment intratubular angiotensinogen and angiotensin II in rats. Am J Physiol Renal Physiol.

[20] Nishiyama A (2012). Mechanisms responsible for the renoprotective effects of renin-angiotensin inhibitors. Yakugaku Zasshi.

[21] Kashihara N, Haruna Y, Kondeti VK, Kanwar YS (2010). Oxidative stress in diabetic nephropathy. Curr Med Chem.

[22] Panda SS1, Bajpai M, Sinha A, Mallick S, Sharma MC (2013). Effect of ipsilateral ureteric obstruction on contralateral kidney and role of renin angiotensin system blockade on renal recovery in experimentally induced unilateral ureteric obstruction. J Indian Assoc Pediatr Surg.

[23] Wolf G (2006). Renal injury due to renin-angiotensin-aldosterone system activation of the transforming growth factor-beta pathway. Kidney Int.

[24] Maki T, Nasa Y, Tanonaka K, Takahashi M, Takeo S (2003). Beneficial effects of sampatrilat, a novel vasopeptidase inhibitor, on cardiac remodeling and function of rats with chronic heart failure following left coronary artery ligation. J Pharmacol Exp Ther.

[25] Wen ZZ, Cai MY, Mai Z (2013). Angiotensin II receptor blocker attenuates intrarenal renin-angiotensin-system and podocyte injury in rats with myocardial infarction. PLoS One.

[26] Volpe M, Savoia C, De Paolis P (2002). The renin-angiotensin system as a risk factor and therapeutic target for cardiovascular and renal disease. J Am Soc Nephrol.

[27] Berk BC (2003). Angiotensin type 2 receptor (AT2R): a challenging twin. Sci STKE.

